# Supplementary material for: Sporadic outbreaks of crimean-congo haemorrhagic fever in Uganda, July 2018-January 2019
Source: PLoS Negl Trop Dis. 2021 Mar 8;15(3):e0009213. doi: 10.1371/journal.pntd.0009213 (PMC7971858; doi:10.1371/journal.pntd.0009213)
Supplement: Attachment — Submitted filename: Response to reviewers.pdf [file pntd.0009213.s001.pdf]

Reponses for the comments made by the reviewers

Comment and suggesttions	Author's response	Line
Reviewer One		
Is your goal to see if risk factors in Uganda are similar to known risk factors in other endemic countries? Or do you want to know whether exposure was due to human contact, tick exposure or livestock exposure so that mitigation measures can be properly allocated?	In Uganda, eight confirmed CCHF outbreaks had occurred between 2013 and 2017. However within 2018, the frequency of the outbreaks had increased hence we investigated why this was the case. In addition, the outbreaks were dispersed than usual, so we wanted to know if the exposures of the case patients were the same or different. When we first investigated possible exposures during hypothesis generation, we found most case-patients had contact with tick-infested livestock rather than contact with confirmed CCHF cases or contact with body fluids from livestock through slaughter or meat handling. We went ahead to prove our hypothesis. It is also important to note, this study involved more confirmed CCHF cases than any other epidemiological studies done before 2018 in Uganda. Findings from this study hence can inform recommendations for control of CCHF infections in Uganda	
I would like further information on how the 14 confirmed cases were identified. Were all 409 suspects tested and there were only 14 confirmed RT-PCR positives?	Yes, all 409 suspects were tested and only 14 tested positive. Uganda's Ministry of Health has a sentinel surveillance system where samples from any suspected case for VHF from all over the country are sent to UVRI for testing. The suspected cases usually meet the standard suspect case definition for VHF included on line 136-138. Samples picked from these cases are tested for multiple VHFs such as Ebola Virus Disease, CCHF, Marburg and Rift Valley Fever among others. Within our study period, 409 samples from suspected cases were sent and 14 were confirmed positive for CCHFv. Note that other VHFs such as RVF were also confirmed	

	within this time however our focus was on the CCHF cases.	
How many medical facilities were included in this surveillance program? How were they distributed within districts? What kinds of facilities were they (referral hospitals, local medical clinics, etc.)?	Uganda's Ministry of Health has a hub system that extends services all over the country to both private and government health facilities. Samples from suspected VHF cases are picked from health facilities where a specified hub rider picks them and sends to reference laboratories i.e. UVRI. In that way, any facility in Uganda with a suspected VHF case can send samples for testing at UVRI. These cases are usually with severe illness and have probably been referred to major hospitals hence most of these samples come from hospitals rather than low-level health facilities.	
Calculating attack rates don't seem appropriate with such small case numbers and likely many missed cases. I suggest analyzing your data regarding age as an odds ratio.	We matched by age therefore this variable was not explored in the case-control. Additionally, there is a significant statistical difference in age according to the analysis.	
I suggest, re-evaluated your stratification methods for calculation of your Mantel-Haenzel odds ratios so that each stratum has larger sample size numbers. Stratification into 14 categories (based on matched case-control subsets, isn't going to give you large enough sample sizes for each strata). Try stratifying by age or sex and see if that significantly changes your adjusted values.	We matched by age and sex. These variables are all statistically insignificant when adjusted by age and sex. This is why we chose to stratify by case-control sets rather than age, sex and sub-county.	
The conclusions are supported by the data presented. Limitations of the study could be further described.	We have further described the limitations as suggested	369-375
The recommendations surrounding tick control are helpful and relevant for this topic and are specific to this region. I'm curious why butchering / slaughtering / handling livestock carcasses and bodily fluids was not investigated as potential risk factors? There are distinct differences with regard to virus exposure between rearing livestock and the act of butchering and it would have been interesting to see if there was a distinction between these two activities in this region. Could this analysis be added? This would	Butchering / slaughtering / handling livestock carcasses and bodily fluids were considered when we were generating the hypothesis. We found that 1/8 case-patients interviewed had handled dead carcasses or raw meat or body fluids i.e. during slaughter/butchering/ abortions. We also found 1/8 had eaten meat with unknown origins making the two less likely exposures of interest. However, we found 8/8 case-patients interviewed had contact with livestock from either within the household or	289

help public health authorities to prioritize interventions around tick exposure as opposed to safe handling of livestock carcasses.	neighborhood. This was the most likely exposure see figure attached. We therefore explored our hypothesis. We have however added the analysis as requested in Table 2. Note: Variables were answered by study participants that reported to interact with livestock 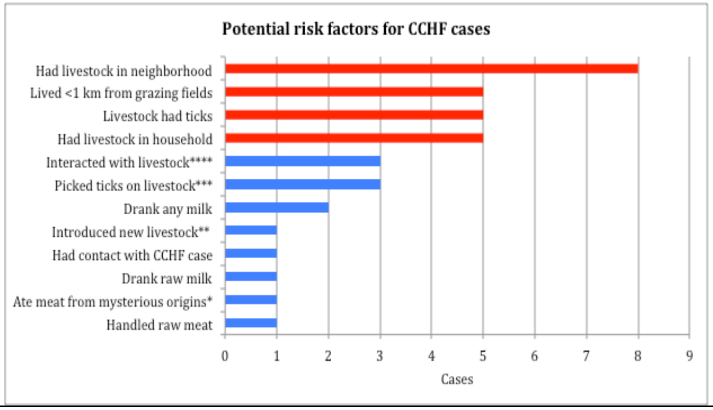	
Line 24 – 25 – Reword - >4 what? (Days or symptoms?)	We appreciate this oversight and it has been corrected	25
Line 27 – Check on distinction between using CCHF and CCHFV throughout. You test for the virus but diagnose the disease.	We appreciate this oversight and it has been corrected	27
Line 35 – 36 – was this difference in attack rates statistically significant?	For age, there was a significant statistical difference with a p0.015. We have included this result into the manuscript.	203-204
Line 46 – What is the One Health aspect of control you are recommending? Tick control for both the environment and animals? This could be clarified.	In our study, we noted that one does not have to possess livestock within their household to be at risk of CCHF. We therefore acknowledge that tick control should be a communal effort rather than individual. For this to be effected, there is need for multiple disciplines in our society to come together and find a solution. For example, acaricide used need to be chosen by National Drug Authority based on scientific findings, political and community heads need to be used to mobilize people towards this cause,	386-390

	entomologists need to continuously understand ticks in Uganda and how they are affected by acaricide resistance, biologists need to develop an alternative environmental friendly way to deal with ticks such as use of predatory birds etc. This is an example of One Health approach recommended.<br><br>We have however clarified our recommendation.	
Line 66 – "caused by a tick-borne virus in the genus Nairovirus, family Orthobunyaviridae"	We greatly appreciate this mistake and have since corrected it accordingly	87
LINE 80 – "other bodily secretions"	We appreciate this edit and have corrected accordingly.	90
Line 93 – reference figure 1 when describing the "cattle corridor"	We have referenced Figure 1 when describing the cattle corridor as suggested.	123
Line 109 – "…occurring between 1 July 2018-30 in a resident of 1 of the 11 affected districts."	We appreciate this edit and have corrected as suggested.	144-145
Line 118 – "We conducted a descriptive epidemiology investigation to collect information on symptoms, demographics, location and timing of infection and associated activities."	We have made these edits accordingly.	153-156
Line 122 – "To identify potential risk factors for infection, we interviewed……"	We have made these edits accordingly.	158
Line 124 – 125 – What was the effective exposure period that you used?	We used the effective exposure period as 1-14 days. We have made this clarification in the manuscript.	161
Line 136 – what do you mean by "population statics"?	There was a typo in this sentence. We meant 'population statistics'.	177
Line 137 – "significance was calculated…"	We have adjusted according to the suggestion	178
Line 145 – Were the non-confirmed suspect case-patients negative on PCR or not tested?	The non-confirmed suspect case-patients were tested and found negative on PCR.	
Line 190 – 193- If you were going to state hypotheses or describe known risk factors for CCHF from other studies, this information would be better placed in the introduction.	We have considered this suggestion and have since included this in the introduction.	128-130

Line 223 -224. This last sentence is redundant.	We have removed the sentence as suggested.	
Reviewer 2		
Methods for logistic and exact logistic regression models are described in the methods but I don't see any results of this described.	We used exact conditional logistic regression with variables that failed to converge during stratification. "Household had grazing fields within 1km" was the variable that was analysed. We show this in the footnote of Table 2	303
I suggest the following sub-headings for results section: 1. Descriptive epidemiology of confirmed CCHF cases, 2. Risk factors for infection with CCHF	We appreciate this suggestion and have made necessary edits	184, 281
The description of results in the case-control portion of the study could be more clearly presented. I suggest presenting the significant risk factors first in a format that focuses on risk. For example, "The odds of living within 1 km of grazing fields for livestock were 18 times higher among CCHF case-patients than controls (ORMH = 18, CI=___; n = 12 (86%) cases and n = 27 (48%) controls lived < 1km)."	We greatly appreciate this suggestion and have made the necessary edits	282-288
Table 1: 5th column title could be "Potential routes of exposure". This column could be presented in more bullet point form than paragraphs. That would consolidate this table.	We have put all content in 5th column in bullet point form as suggested.	231
Was being within the cattle corridor a significant risk factor? I suggest adding this to your table.	One of the criteria for matching cases and controls was by i.e. sub-county. The 'cattle corridor' stretches over large area involving multiple districts hence no variation in this aspect can be seen among case-patients and control-persons.	
Public health relevance is addressed in the study.<br>My main concern with this manuscript is that the authors have indicated the need to implement tick control as a method to	We can only infer to accarcide resistance in other tick species other than Hyalomma ticks. This due to minimal studies targeting resistance in Hyalomma ticks specifically. We have clarified this	347

reduce the burden of CCHFV infections and make reference to possible increased tick resistance due to acaricide control. However although increased tick control would be recommended nowhere in their manuscript do they actually mention that CCHFV is transmitted by ticks belonging to the genus Hyalomma, which are considered to be the principal vectors. although the virus has been isolated from some 30+ species of tick, the role of these ticks as vectors is not clear. In addition the paper they refer to for increased tick resistance was based on data mostly for Rhipicephalus species (no Hyalomma were identified in the paper). hence although the indiscriminate use of acaracides may have influenced hyalomma populations there is no evidence from the manuscript cited to support this. this needs to be made clearer in the manuscript.	concern within the manuscript.	
what tick species occur in the districts investigated? was there any information on the tick species described in this study? if no information then please include this as a limitation of the study.	We have added this to our limitations. We could not determine the tick species. However based on research, Rhipicephalus species are more prevalent in South Western Uganda.	370-376
Line 251: the case living in an urban area may also have been exposed to infected blood during slaughter of animals as religious festivals and so this sentence regarding tick exposure is not justified and considering there was evidence of tick-bite.	Despite having no evidence of a tick bite since she was deceased, we were certain that this particular case had no role in slaughter or handling of animal carcasses or raw meat within the effective exposure period. This case did not even participate in preparation of any of the food consumed during the celebrations. This is based on the qualitative interactions we had with the family.	
Lines 6 to 7: Please correct, CCHF is caused by Crimean-Congo hemorrhagic fever orthonairovirus a member of the Orthonairovirus genus and the family Nairoviridae	We have corrected this accordingly.	87
Line 73: Crimean-Congo haemorrhagic fever orthonairovirus (CCHFV) is a tick-borne virus found in Africa, Asia, eastern Europe and the Balkans. I suggest adding : The geographic	We have included this statement in the manuscript as suggested.	95-97

distribution of the virus correlates with that of ticks belonging to the genus Hyalomma, which are considered to be the principal vectors.		
Line 76-77: the authors state that The incubation period for CCHF in humans depends on viral dose, genetic factors and immune status of the host, and route of infection (6, 7). it is accepted that the incubation period does vary with source of infection, that is tick bite versus exposure to infected tissues, but the role of viral dose and genetic factors is not so clearly defined. I am unsure what is referred to when saying immune status influences incubation period.	Yes, the genetic and immune status are not yet backed by science however this is suggested in this study 'Crimean-Congo Hemorrhagic Fever: Tick-Host-Virus Interactions' https://www.frontiersin.org/articles/10.3389/fcimb.2017.00213/full I have however deleted this statement from the write up.	
Line 78: authors state that Incubation is typically 1-9 days following a tick bite, and 5-13 days following contact with infectious fluids. it would be more accurate to indicate that incubation period is usually 2 to 3 days following tick bite although occasionally longer the incubation period is typically short after tick bite.	World Health Organisation states this incubation and we had heeded to it because we use the IDSR guidelines in surveillance of VHF in Uganda.	
Please provide references for all instances where previous cases in Uganda are referred to.	We could not obtain all this information as requested. We however could have misunderstood the comment.	
The authors have submitted a paper that will contribute to our knowledge of CCHF infections in Uganda and hence is an important paper but I suggest some of the limitations of the study especially with regard to the lack of information on tick species be considered.	We have added a section on study limitations	370-376
