# Supplementary material for: Sporadic outbreaks of crimean-congo haemorrhagic fever in Uganda, July 2018-January 2019
Source: PLoS Negl Trop Dis. 2021 Mar 8;15(3):e0009213. doi: 10.1371/journal.pntd.0009213 (PMC7971858; doi:10.1371/journal.pntd.0009213)
Supplement: Attachment — Submitted filename: Response to Reviewers.doc [file pntd.0009213.s002.doc]

Reponses for the comments made by the reviewers

| Comment and suggestions | Author’s response | Line |
| --- | --- | --- |
| Reviewer One |  |  |
| Overall, the methods were appropriate for the data and to address the stated objectives. There is a discrepancy between descriptions of the case-definition between the main text and the abstract. One states all cases were confirmed by PCR and the other says PCR or IgM serology. This is an important distinction that should be clarified. Including only cases that tested positive by PCR would be stronger. | We have adjusted the statements as suggested. UVRI uses PCR and IgM serology tests however we have revisited the reports submitted and all the cases included were confirmed using PCR. | 28, 120 |
| "Exact conditional logistic regression was used with variables that failed to converge during stratification." I'm not following this part. There shouldn't have been model convergence issues with calculating M-H Odds ratios. Is this description referring to your method for calculating the crude Odds ratios that you report in the results? If so, I would state it like that. | We calculated the crude odds ratio using simple 2x2 analysis. However, during stratification, some cells had zero counts hence we used exact conditional logistic regression to derive the M-H Odds Ratio. We have added a statement about how the crude odd ratios were derived. | 147-148 |
| The population is clearly described and appropriate for the hypothesis being tested. The number of confirmed cases is small but given the type of study, the analyses and the use of 4 matched controls per case was appropriate. There are no concerns about ethical or regulatory requirements being met. | We greatly appreciate this comment. |  |
| Table 2: The sample sizes for individual acaricides in the table is small and no statistics at the individual level are provided, so I would remove these. Frequency of spraying livestock also doesn't seem to fit as a "risk factor" for developing CCHF in this table because no analyses of them as risk factors are provided. | We have removed the section on acaricides as suggested since no statistics at individual level are provided and since it does not fit as a risk factor for developing CCHF. | 213-214 Table 2 |
| For the "Butchering meat" and "Slaughtering animals" factors can those be categorized as something other than N/A's. Weighted correction methods such as the Haldane-Anscombe correction could be used to calculate odds ratios when one of the categories is 0. For some risk factors not all of the cases are represented (i.e. slaughtering animals only has data from 10 cases). Was data not collected for some of these cases on these topics? | In our questionnaire, we would ask if the person interacted with livestock before we would ask the follow up questions on how they interacted with livestock. We determined that 10 cases interacted with livestock while other did not. Therefore, the follow-up questions on “butchering meat” and “slaughtering animals” were asked to only those 10 cases. This variable is part of the Table 2. We initially had removed the variables derived from “interacted with animals” since it was not statistically significant and was a less likely exposure of interest during hypothesis generation. In the previous review, the reviewer was curious why we did not investigate butchering / slaughtering / handling livestock carcasses and bodily fluids as potential risk factors. We had added it back to the manuscript to show the values we got. We honestly do not expect these variables to be informative on risk factors in this study which is why we have decided to omit them again. However, if the reviewers still suggest that they are an important part of the study, we will gladly re-add them into the manuscript and explore how best to analyse to include them. For more clarity, we have added a description on what we meant by “interacted with livestock” at the bottom of Table 2. | 213-214 Table 2  216-217 |
| Study Limitations: I don't think you need to state these. You did a nice risk factor study regardless of whether your original objectives were met, and this section feels a bit like it comes out of the blue because it wasn't mentioned earlier as an objective of the study. | We added this because one of the reviewers suggested in the previous review that we lacked information on tick species and it was a study limitation. We had indeed collected blood and tick samples from livestock found within the households with the aim of verifying if they were infected with CCHFv and identify the tick species as well. Unfortunately, no further analysis was done due to resource constraints which is why we had no information on ticks found in households. We have however restructured the paragraph to better communicate the limitation. | 280-283 |
| Conclusions are supported by the data presented. The conclusion section could take more of an active voice as you are making these recommendations to the people reading the paper in addition to the people you have already informed on the recommendations. The public health relevance is clearly addressed. | We appreciate this comment and have re-phrased the statements accordingly. | 285-306 |
| The study limitations could mention the small number of cases that met the case definition. The discussion of development of resistance to acaricides amid inconsistent use is interesting and highly relevant to future public health interventions. Perhaps a short discussion of recommendations for future research in this area, to identify specific acaricides in which ticks have developed resistance to in Uganda is worth mentioning. | We have added the small number of cases as a study limitation and showed future research suggestions. | 279-280  302-304 |
| Throughout: double check the use of CCHF vs CCHFV to make sure you are referring to the disease or the pathogen the way intended  Line 27: should be "CCHFV"  Line 72: use CCHFV  Line 77: CCHFV  Line 80: CCHFV  Line 82: CCHFV  Line 117: CCHFV  Line 119: CCHFV  Line 153: CCHFV-positive  Line 220: CCHFV  Line 266: can use CCHF virus or CCHFV but be consistent throughout paper | We appreciate this observation and have revisited the entire manuscript to ensure that CCHF and CCHFv are correctly used. | Entire manuscript |
| Line 37: missing an "=" sign for P value | We appreciate the oversight and have since corrected this. | 38 |
| Line 45: I like that you are calling out the One Health approach here but it's a bit vague what the context is. Are you meaning that preventive measures for tick control should be taken for both livestock and humans to lessen human exposure to tick bites? A sentence of clarification would help here. | Animal health in Uganda is under MAAIF and livestock farmers have the sole responsibility to manage tick infestation on their animals. Acaricides are regulated by National Drug Authority which an autonomous authority under the Ministry of Health and plays the crucial role in control of acaricides on the market. The challenge is CCHF does not impact animal health because it generally doesn’t affect animal health however Ministry of Health does not have a direct role in tick control or how farmers spray their animals. This makes tick control complex in Uganda. However, there are other tick-borne diseases that are a concern to animal health. Therefore, all these institutions can come together towards control of ticks in turn implementing CCHF prevention and control. We have added a statement to clarify our suggestion in both the abstract and recommendations. | 45-47  292-308 |
| Line 70: "or from one human to another" | We have adjusted this. | 70 |
| Line 71: the term "wild livestock" sounds contradictory; change to "domestic livestock and wild ungulates", or "wildlife" | We appreciate the suggestion and have rephrased accordingly | 71 |
| Line 74: "important predictor of virus distribution" | We have adjusted according to the recommendation | 75 |
| Line 83: should be "other bodily secretions". | We appreciate the observation and have adjusted accordingly | 84 |
| Line 185: should be "did not report tick bites" | We appreciate the observation and have adjusted accordingly | 188 |
| Table 1: Use periods at the end of each bullet or don't use them but be consistent throughout table. | We appreciate the suggestion and we have chosen to remove the use of periods in the table | 197-198 Table 1 |
| Line 202: "In the case-control study, we found that people living within 1 km of grazing fields for livestock were 18 times more likely to be CCHF case-patients than controls" | We have rephrased these statements as suggested. | 205-211 |
| Line 261: change to "at risk for CCHF outbreaks". | We have edited this accordingly | 264 |
| Line 268: Use alternative word than "interrupting", such as "preventing" | We have used “preventing” instead as suggested | 271 |
| Line 276: "similar strains of CCHFV" | We acknowledge this suggestion however we have since rephrased the sentence. | 280-283 |
| This research identifies the most common risk factors associated with CCHF outbreaks in Uganda which has implications for implementation of public health measures to prevent this disease. It's valuable to know that tick bites as opposed to exposure to bodily fluids from livestock appears to be the major driver of cases in Uganda. The case information is clearly presented. Table 2 could use some further consideration on what should be presented as a risk factor as described above. | We appreciate your comment regarding our study. We have addressed the comments on Table 2 above as suggested. | 213-214 Table 2 |
| Reviewer 2 |  |  |
| No comments or suggestions to address | We appreciate taking time to review the manuscript. We had no adjustments to make from Reviewer 2 |  |
